# Compact N-Band Tree-Shaped Multiplexer-Based Antenna Structures for 5G/IoT Mobile Devices

**DOI:** 10.3390/s20216366

**Published:** 2020-11-08

**Authors:** Amélia Ramos, Tiago Varum, João N. Matos

**Affiliations:** 1 Instituto de Telecomunicações, Campus Universitário de Santiago, 3810-193 Aveiro, Portugal; tiago.varum@ua.pt (T.V.); matos@ua.pt (J.N.M.); 2 Universidade de Aveiro, Campus Universitário de Santiago, 3810-193 Aveiro, Portugal

**Keywords:** frequency multiplexed, IoT, millimeter-waves, multi-band, n-band antenna, antenna as a sensor

## Abstract

This paper presents a simple, compact and low-cost design method that allows one to obtain low-profile multi-band antennas for the overcrowded future generation networks, which are widely versatile and very heterogeneous in the K/Ka bands. The proposed antennas comprise *n* radiating monopoles, one for each of the desired operating frequencies, along with a frequency selective feeding network fed at a single point. This concept enables a single antenna to be shared with different radio-frequency (RF) frontends, potentially saving space. Typically, with *n-band* structures the biggest challenge is to make them highly efficient and here this is assured by multiplexing the frequency, and thus isolating each of the monopoles, allowing the design of scalable structures which fit the 5G applications. Based on the vision proposed here, a dual-band and a tri-band structures were built and characterized by their main parameters. Both prototypes achieved peak efficiencies around 80%, with adequate bandwidths and gains, as well as great compactness.

## 1. Introduction

It is clear that the global tendency of exchanged data traffic is growing exponentially and, with it, the number of interconnected devices, pushing existing systems to their limits. In addition to the increasing traffic on each network, the variety of the services supported is increasing too, which naturally gives rise to great concerns about energy consumption. In this sense, 5G wireless systems must fulfil three main requirements: (i) have high throughput; (ii) serve many users simultaneously; and (iii) have less energy consumption [1], the latter being probably the biggest driving force of 5G. Associated with this, the high user mobility will force new antenna designs and new concepts to be implemented [2].

In addition, migration to mmWaves becomes mandatory, because despite implying higher propagation issues, it is probably the most effective way to achieve the necessary bandwidth [3]. In these frequencies, new challenges arise, given the reduced dimensions, however, there is an inherent opportunity to produce compact solutions and an increasing demand for robust multipurpose dual-band or multi-band antenna systems for 5G applications. Today, mobile devices comprise a wide range of applications and features, most of them involving several communication frontends, and also multiple antennas, requiring space, which can create problems such as the coupling between them.

**Figure 1:**
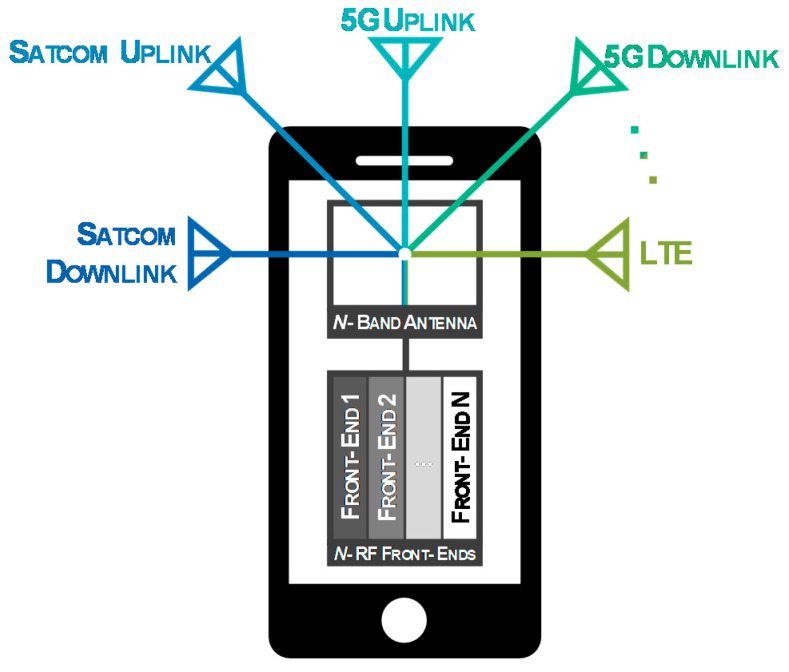
Single N-Band Antenna Shared by Multiple Frontends.

A possible solution to combat the lack of available space resulting from this growing incorporation of communication systems, is to develop a single antenna, operating in each frequency band, and shared by the various communication systems. Figure 1 clarifies the scenario referring to a mobile device where a single antenna structure interacts with multiple radio-frequency frontends, which can be one of the main characteristic nowadays, since even a common smartphone gathers multiple antennas, enabling the connection with the most varied services.

There are several ways to design an antenna that meets these requirements. One is through the bandwidth, i.e. producing an ultra-wideband antenna, however, it this (obtaining a band that includes all the necessary frequencies) is an almost impossible condition, especially given the disparity of frequency bands used. In addition, isolation between bands and frontends would not be guaranteed, decreasing the efficiency.

Another possibility is to develop antennas resonating in different bands, which are the commonly named multi-band antennas. This option consists of a process with a high degree of randomness, since in most cases this is done at the expense of deformations of the radiating structure, presenting little freedom to the control of the antenna’s properties. There is also the group of reconfigurable antennas (in this case, in terms of frequency), although these antennas are much more complex because they are active and require additional control to select the operating frequency. In addition, these antennas use switches in the form of discrete elements, which consume energy. Due to all this, an alternative design is proposed.

This paper is divided into 6 sections, starting with this introduction. Section 2 presents a careful revision of the related works, focusing on multi-band antennas and in frequency-selective feeding structures. Then, in Section 3 the design principle is presented, clarifying the concept proposed. Section 4 contains all methods and presents the structures developed, each in a different sub-section. The results, both measured and simulated can be found in Section 5, as well as the prototypes built. Finally, major conclusions can be found in Section 6.

## 2. Related Work

In this way, several authors have tested the feasibility of reducing the number of antennas in each equipment and thereby saving space. In [2] a structure based on a dual-band slot is proposed and is designed to operate at 28/38 GHz. Along with the operating frequencies, this antenna exhibits adequate bandwidth values, as well as adequate gains for incorporation in a 5G mobile device. Despite the interesting results, this work lacks measures and the authors end up defending their proposal by comparing CST with HFSS.

In [4] another dual-band antenna in a massive MIMO arrangement is described. This structure is based on a series-fed antenna array and one can denote high gains (of more than 12 dB at each band) and good radiation patterns, however, once again, only simulation values are shown.

On the contrary, in [5] the measurement results support the work on a substrate integrated waveguide (SIW) antenna array for the Ka-band. There, authors implemented a linear array to improve the overall antenna’s performance and they were able to achieve satisfactory bandwidths (lower than 5%) and proper gain measurements (around 5 dBi). The authors claim that by using the presented structure, multi-band antennas can be designed, but no implementation is shown to prove this statement.

Additionally, a dual-band dual-circularly-polarized antenna operating in the Ka-band is presented in [6]. The work is supported by simulated and measured results, however, a mediocre correspondence can be denoted between simulations and measurements. The gain, both in the uplink and downlink bands, is improved when the radiating element is implemented into an array, however measurements regarding axial ratio are poor, the complexity is huge and both the antenna’s scalability and the robustness are questionable.

When it comes to tri-band antennas, neither the microstrip patch antenna presented in [7] nor the antenna array with defected ground seen in ref. [8] show any measures confirming their respective simulations. Both structures have resonances in the mmWaves region, along with suitable bandwidths and gains. In [7] the efficiency values shown are quite high, whereas in [8] these values are not mentioned.

Additionally, a scalable structure can be found in [9]. It proposes a compact dual-band and small slot antenna without compromising its performance. Measurements show good correspondence with simulations, as well as good gain, considering the typical values for slot antennas. Apart from these good results, one could argue that the major contribution of the work is its scalability, since authors defend that by adding more slots to the structure the desired multi-band behavior can be achieved. Nevertheless, the structure proposed operates at 2.4/5.2 GHz, very low frequencies for the 5G context.

Recently, an aperture-sharing integration methodology implementing a 3.5/28 GHz antenna with mmWave beam steering capability was proposed [10]. The main concept is to share the aperture of a linear 28 GHz array, comprised by four separately fed dipoles, with a 3.5 GHz dipole antenna. Favorable results were obtained regarding a stable mmWave beam at different scanning angles, meanwhile with broad impedance bandwidths in both operating bands (over 20%). Nevertheless, only one of the resonating frequencies suits the mmWave spectrum region and the overall size of the structure is close to the size of a single 3.5 GHz dipole antenna, which, in many of the future applications may be inappropriate.

In [11] an interesting approach is conducted on using the half-mode substrate integrated waveguide (HMSIW) technique to design low-profile cavity-backed multi-band antennas. Authors designed single, dual and triple band structures to validate the multi band responses which indicated favorable results on the radiation patterns’ stability and the front-to-back ratio. Moreover, the radiation efficiency being higher than 80% at the operating frequencies is a quite encouraging result to test this concept of introducing U-shaped strips outside the aperture of an HMSIW cavity in the mmWaves region of the electromagnetic spectrum, since the tests made were at the C-band.

In [12], an architecture whose main objective is to solve the bandwidth limitations of phased arrays was proposed. The suggested design includes five printed quasi-Yagi antennas, which should be placed in the upper edge of a mobile device. Their placement, and the orientation of the active element and the directors are crucial to solve such bandwidth limitations. With the suggested configuration and by not using phase shifters and simply switching the feeding to one of the quasi-Yagi elements it is possible to scan the desired areas. However, this switchable antenna system results in a physically larger setup, since only one antenna is used at a time, which when compared to the phased array is a disadvantage, as in these structures, antenna area can be saved, as the whole aperture is exploited.

Another structure which explores alternative designs is presented in [13]. Considering the advantages of omnidirectional radiation patterns in communicating regardless of direction, a modified fork-shaped microstrip monopole antenna with a probe feed line shows wideband and multi-band characteristics. Here, the impedance bandwidth is improved by designing a dual-triangle portion of the ground plane, yet the resonant frequencies are quite low, suitable for example for the GSM band. 

Regarding the emerging MIMO systems, in [14] a MIMO antenna system for multi-band 5G (mmWave) and wideband 4G application is shown. This structure works at triple bands (28, 37 and 39 GHz) for 5G and the wideband (1.8–2.6 GHz) for 4G. Each one of the MIMO elements consists of a slot in the ground plane and two microstrip feeding ports in the top layer (the isolation is also enhanced by using a low pass filter). Indeed, this design can work as a tapered slot antenna for 5G, covering 27.5–40 GHz or as an open-ended slot antenna for 4G covering 1.8–2.6 GHz. However, as noted, in the 5G band one denotes wideband operation, instead of multiple resonances at the frequencies of interest.

One of the major challenges of a multi-band antenna structures is its efficiency, a specification that gains further importance when operating in the mmWave region. The major contribution of this work is the microstrip feeding arrangement, which allows to section the antenna into *n* frequency-selective parts. In the literature, other techniques can be found to improve the feeding network’s efficiency.

In [15], an antenna array consisting of five radiating elements is designed and measured, operating at the 2–4 GHz range, achieving a beamwidth around 24 degrees. The frequency selective feeding network delivers the signal to the selected elements at 2*f*_0_ and gradually switches the signal between elements as the frequency decreases to *f*_0_. The major advantage of this strategy is an almost constant beamwidth over a broad frequency range.

A similar concept is presented in [16] where a six-element antenna array operating in 1.75–3.5 GHz frequency range is seen. In this report, the feeding network uses a directional filter where the adjustment of coupled-line sections allows for the flexible selection of transmission coefficients. This structure permits a constant beamwidth in an octave frequency range the signal is redirected from the center elements to the outer elements. In the end, this feeding network’s achievements are reached at the expense of a single directional filter and equal split power dividers.

More recently in [17] an interesting approach on multi-band filtering slot antennas is proposed. There, authors realize a duplexing and filtering antenna by integrating a multi-band antenna and the multimode resonator. The work is validated since three antennas were designed, fabricated and measured. Although the correspondence between simulations and measurements is quite satisfactory, neither the antenna’s scalability is a priority nor the operating frequency is suitable for the future generation of mobile communications. Above all, the concept proposed in [17] requires the usage of two feeding ports, which differs from the goal of this work which lies on having a single antenna, fed in a single point, capable of interacting with multiple RF frontends, isolating each frequency.

All of these suggested frequency selective networks are associated with higher complexity in the structure’s design and fabrication. They demand for additional components and the antennas were designed for lower frequencies than what is expected within the 5G context. The work presented here defends an innovative concept where neither the manufacturing is compromised nor the feeding network imposes the usage of any additional components.

In this sense a new concept to design a structure for a multi-band antenna operating in the K/Ka bands is proposed. The design idea lies on sectioning a n-band antenna into *n* parts, having each section properly isolated (in frequency) in order to maximize the efficiency, and that is achieved by applying the adequate impedance matching as it will be explained later. This frequency multiplexing concept represents the main difference between the state-of-the-art studied and the structures proposed in this report. With this alternative it is possible to several antennas in a single structure with a single feeding point. Two prototypes were built and tested representing the cases of a dual and a tri-band antenna, ensuring the scalability to other resonances.

## 3. Design Principle

Devices which operate in multiple bands are highly interesting, given the wide and varied protocols that they are equipped to operate. To exemplify, the architecture of a current smartphone includes capabilities to operate with different communication technologies such as Wi-Fi, Bluetooth, GSM, 3G/4G (LTE), GPS, NFC, among others. In addition to multiple radios, these devices require several antennas, increasing interferences and couplings, and it is an ever more difficult task to accommodate them due to the reduced space available. It is in this context that a new concept for designing a multi-band antenna, is inserted.

**Figure 2:**
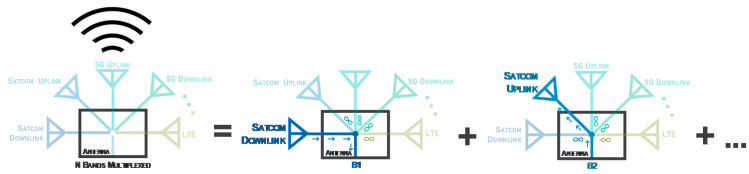
Schematic of the Main Design Concept.

Figure 2 presents a schematic example of a multi-band antenna for modern terminals. As noted, the antenna structure in the example operates in the downlink and uplink bands of 5G, SATCOM and LTE. In the illustration, instead of five antennas, with this concept it is possible to design a single antenna operating in all desired bands, with only one feeding point.

Being an antenna shared by different radios, it is important to assure isolation between all bands, that is, that each RF frontend receives/transmits information using only its respective resonating element. The project begins with the design of all *n* resonant antenna elements, one for each operating frequency. After having the *n* radiating elements, the feeding structure ensures the high global efficiency of the antenna, forcing theoretically infinite impedances in the operating bands of neighboring elements, clearly conducting the signal towards/from the respective resonant element.

As an example, Figure 2 can be analyzed in depth, where in the middle schematic the user wants to receive data in the SATCOM downlink band, and thus the path is clear for the respective frequency band B1, and for all the other operating bands the impedance seen will be infinite. This concept is scalable for the *n* different bands of the antenna. In short, this concept allows to design a *n-band* structure as efficient as the summation of several resonant antennas in each of the *n* bands.

## 4. Methods and Structures

The great advantage of having the prototype composed of *n* sections is to guarantee that for each operation frequency, the input impedance is only imposed by the respective resonant element. This leads to a very efficient feeding network, in line with the challenging requirements of 5G, massive IoT and the future satellite communications.

Nowadays, wireless communications systems are mainly equipped using printed antennas [18,19] mostly due to their low cost and ease of fabrication. Another important aspect is the ability to produce various structures, with unlimited design shapes and typologies. Bearing in mind the need to ensure communication regardless of direction, monopole and dipole-based structures present interesting advantages.

**Figure 3:**
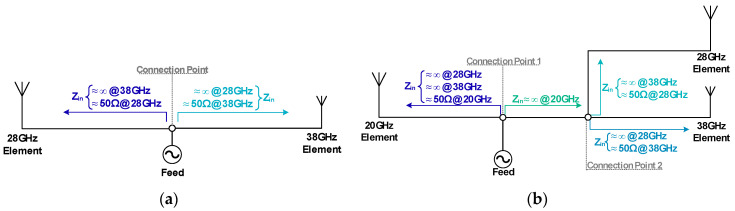
Schemes Proposed for the (**a**) Dual-Band and (**b**) Tri-Band antenna.

In order to verify the practicability of this concept, it was decided to demonstrate it, designing two prototypes, a dual band and a tri-band antenna. Starting with the dual-band model, it is composed by two printed monopoles, each resonant at a different frequency, 28 GHz and 38 GHz respectively. Additionally, confirming the scalability of the concept, a tri-band antenna was developed, and for this, a radiator at 20 GHz was added. Both schemes are presented in Figure 3, clarifying the arrangement and the impedances expected in different parts of the structure.

The key aspect of these antennas is the feeding structure. The sectioning of this antenna into different parts lies on the existence of the connection points presented in Figure 3, thus, to scale this concept to a *n-band* antenna, *n-1* connection points are required. These connection points represent the division of the feeding network. In the example, for a dual-band antenna, there is one single connection point, however, for the tri-band structure, two connection points are required, and so on.

The resulting parts of the overall antenna have an adequate matching at the resonant frequency of the respective radiating element, while simultaneously showing a close to infinite impedance at the frequency of the opposite radiating element. Therefore, at each resonant frequency, a parallel association of infinite impedances with a characteristic impedance of interest is observable.

Clarifying with Figure 3a, at the connection point and looking at the 28 GHz element: at the resonant frequency, 28 GHz, an adequate matching is verified ensuring that it works properly, and, at the same time, the feeding network warrants that at 38 GHz a very high impedance appears. These principles are applied symmetrically to the opposite side of the structure.

Both antennas proposed are designed over a single layer of the dielectric substrate Rogers RO4350B, which main properties include a dielectric constant *ε*_r_ = 3.48, thickness *h* = 0.254 mm and dissipation factor of tan(δ) = 0.0037 @ 10 GHz. All simulations have been carried out using the electromagnetic simulator software which is the Computer Simulation Technology Microwave Studio Suite (CST-MWS).

### 4.1. Dual-Band Antenna Design

**Figure 4:**
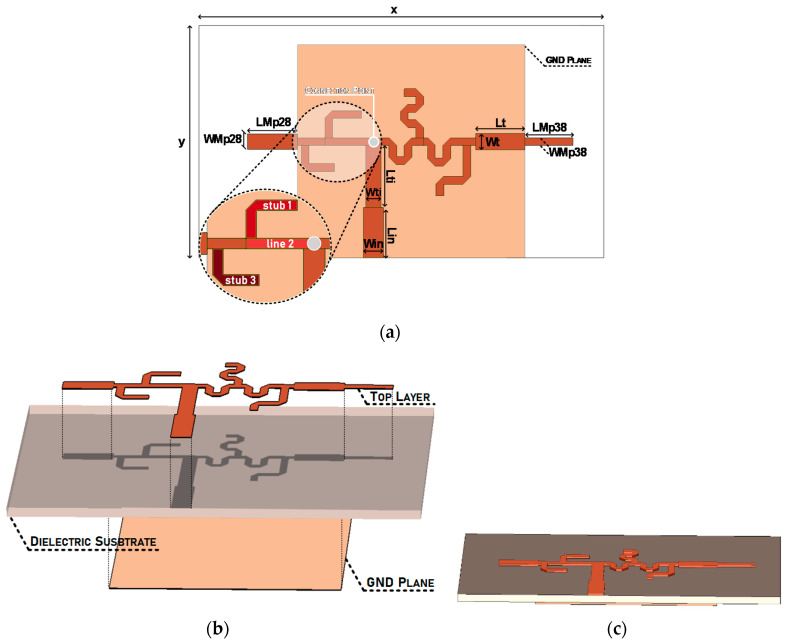
Dual-Band Antenna: (**a**) Design Parameters, (**b**) Layers Identification and (**c**) Final Structure.

Figure 4 identifies the main design parameters of the dual-band structure. Two monopoles with dimensions *LMp28 × WMp28* and *LMp38 × WMp38* were designed. A 50 Ω impedance microstrip line, along with a quarter-wavelength transformer lead the input signal to the connection point referred above. Additionally, in order to maximize the structure’s compactness, meander lines were used for the greater stubs.

From the connection point up to the monopoles, there is a group of microstrip lines which guarantees the adequate matching (at the desired frequency) and the close to infinite impedance at the adjacent operating band.

The acceptable matching is achieved by the colored identified lines highlighted in Figure 4a. These lines’ dimensions and placement were implemented bearing the main concepts of microwave propagation. Starting with the open-ended stub named *Stub 1*, which has a 90° electrical length of 90° at 38 GHz, it turns infinite impedance into a short circuit. Naturally, *Line 2*, having the same electrical length at 38 GHz, converts the above-mentioned short circuit once again into a very high impedance at 38 GHz.

Through this pair of lines, the first requirement at the connection point is guaranteed: an (close to) infinite impedance at 38 GHz. However, these lines surely also influence the impedance in this part of the antenna, at 28 GHz. Therefore, the third line, *Stub 3*, was placed to compensate for this undesired influence, ensuring the impedance matching at 28 GHz.

Symmetrically, on the other side of the connection point, there is an open-ended stub, immediately followed by another stub, both with an electrical length of 90° at 28 GHz, enabling the open circuit in the connection point at 28 GHz. Subsequently, a third stub once again compensates the influence in the antenna matching observed at 38 GHz.

After bearing all theoretical propagation considerations, the dual-band antenna optimized design parameters are presented in Table 1 and the feeding network line width is constant throughout the antenna (0.2 mm), with the exception of the quarter-wavelength transformer (*Lti × Wti)* and the 50 Ω input line (*Lin × Win*), connected to the single feeding point.

### 4.2. Tri-Band Antenna Design

**Figure 5:**
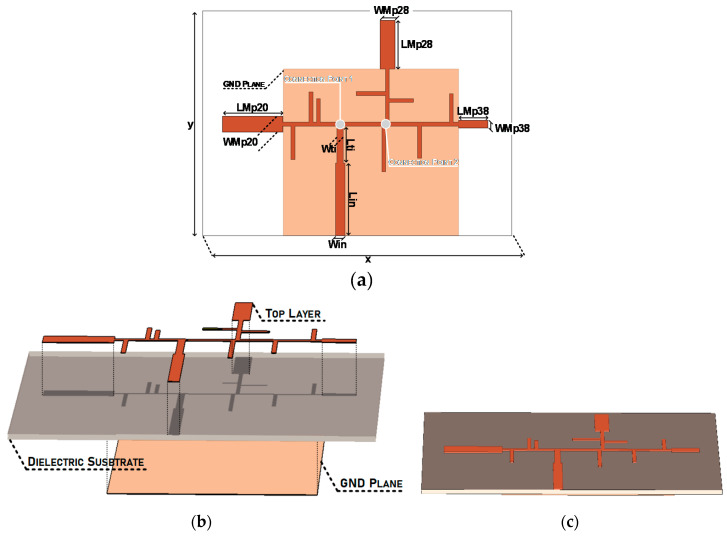
Tri-Band Antenna: (**a**) Design Parameters, (**b**) Layers Identification and (**c**) Final Structure.

Using the same method as before, and considering both the impedance requirements showed in Figure 3 and the design principle of Section 3, the tri-band antenna was designed over the same dielectric substrate used for the dual-band structure and its main design parameters are presented in Figure 5.

The three resonant elements are easily denoted in Figure 5 through their associated design parameters *LMpx × WMpx*. The main design parameters are listed in Table 2. All microstrip lines designed have 0.2 mm width, apart from the 50 Ω input line (*Lin × Win*) and the quarter-wavelength transformer (*Lti × Wti*), as in the dual-band example. In this case, the final version of the monopoles has lengths equivalent to 0.4λ_d_, 0.45λ_d_ and 0.37λ_d_, regarding the 20, the 28 and the 38 GHz monopoles, respectively.

## 5. Results and Measurements

**Figure 6:**
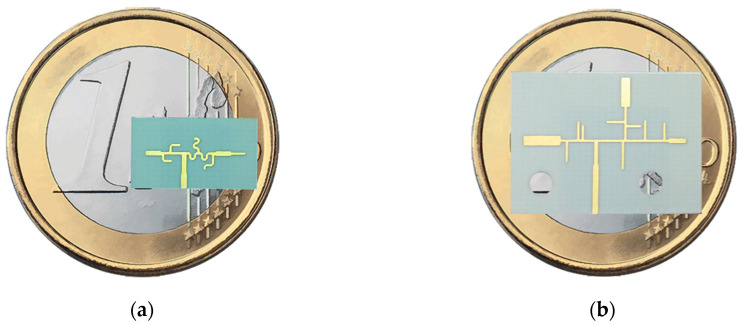
Prototypes Built: (**a**) Dual-Band and (**b**) Tri-Band Antennas.

The previously described prototypes were simulated and built and they are shown in Figure 6. It is important to mention that regarding the tri-band structure, Figure 6b presents a connectorized version of the antenna, where the length of the 50 Ω input feed line is excessive and just needed to place the connector. The antennas were measured, and the main results obtained are presented throughout this section.

**Figure 7:**
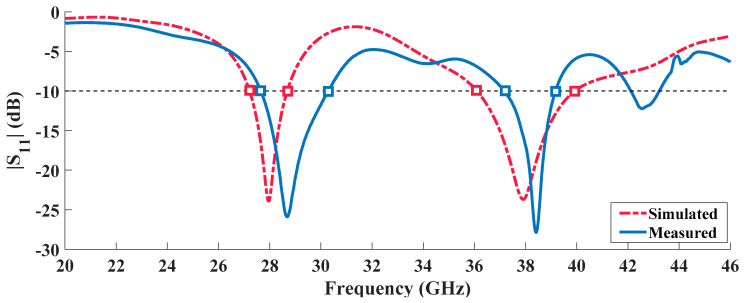
Simulated and Measured Reflection Coefficient of the Dual-Band Antenna.

Figure 7 exhibits the simulated and measured results of the reflection coefficient for the dual-band structure.

For this prototype, a good impedance matching was achieved, with both curves presenting a similar behavior despite some frequency shifts of 680 MHz and 410 MHz from the desired 28 and 38 GHz, respectively. These small discrepancies seen might be justified either by slight construction imperfections (which is something natural given the antennas’ small size). Nevertheless, the measured values of S_11_ at the operation frequencies are −13.64 dB and −16.94 dB, respectively, making of these results quite satisfactory.

Regarding the bandwidth, and considering the S_11_ < 10 dB criteria, the prototype achieved an operating band of 2.55 GHz [27.54–30.09 GHz], representing 8.8% bandwidth around 28 GHz. On the other hand, around 38 GHz, a 1.98 GHz [37.2–39.18 GHz] band was reached, meaning 5.2%. Table 3 summarizes the measured and simulated results of the dual-band radiating element.

**Figure 8:**
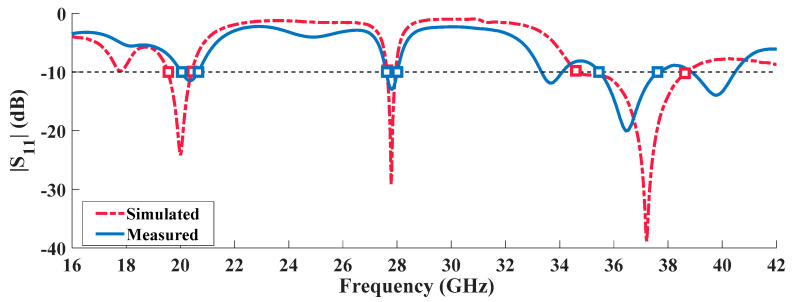
Simulated and Measured Reflection Coefficient of the Tri-Band Antenna.

The second prototype built proves the concept here proposed in a tri-band antenna. Figure 8 identifies a reasonable impedance matching between simulated and measured results in respect to the reflection coefficient, since the minimum value for S_11_ occurs at the frequencies of interest, despite the slight frequency shifts around 20 GHz and 38 GHz.

These minor mismatches can be once again justified by the same reasons presented for the previous prototype: construction imperfections, or dielectric permittivity variations. Regarding the first operation band (at 20 GHz), the antenna shows a measured bandwidth of 0.62 GHz, equivalent to 3%. At the other operation frequencies, 0.4 GHz and 2.16 GHz are seen at 28 and 38 GHz respectively. Table 4 summarizes the measured and simulated results of the tri-band radiating structure.

Through the reflection coefficient of the latest prototype analyzed it is possible to confirm the adequacy of the prototypes built and more importantly the principle of design proposed seems to guarantee the impedance matching.

**Figure 9:**
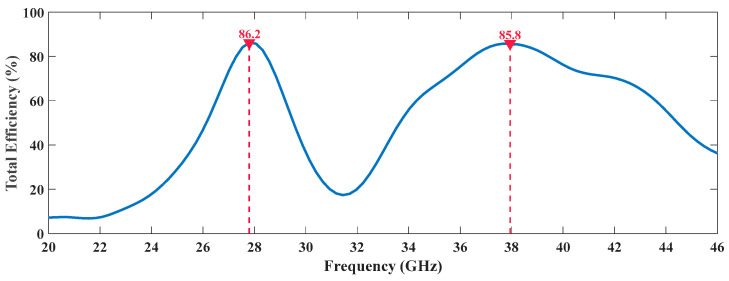
Efficiency Variation Over Frequency of the Dual-Band Antenna.

**Figure 10:**
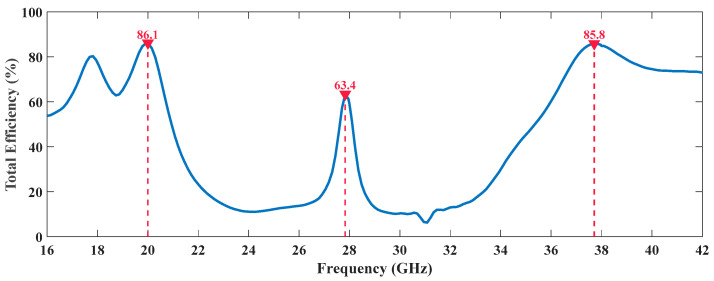
Efficiency Variation Over Frequency of the Tri-Band Antenna.

Other aspect that sustains the appropriate performance of these antennas in 5G and the massive IoT scenario is the information about the antennas’ efficiency, which is presented in Figure 9 and Figure 10, regarding the dual and the tri-band radiating structures, respectively.

Figure 9 presents the simulated efficiency of the dual-band antenna over the frequency. Naturally two local maximums are highlighted. These maximums are according to expected since they appear adjacent to the frequencies of operation. Their magnitude is higher than 85% in both operating frequencies, an important value given the structure’s compactness and size.

Similarly, for the tri-band antenna, there are clearly three local maximums in Figure 10, which are once again, near the frequencies of interest, in this case 20, 28 and 38 GHz, confirming the proper functioning of the antenna.

**Figure 11:**
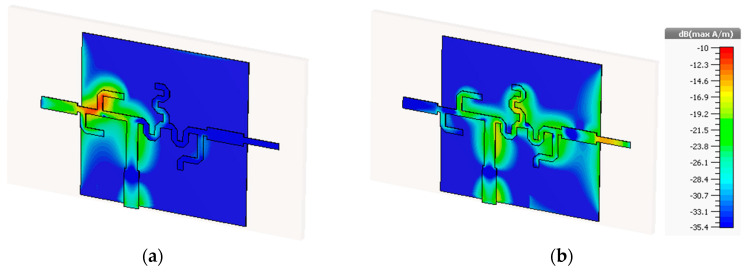
Surface Current Distribution at (**a**) 28 GHz and (**b**) 38 GHz.

Besides the efficiency peaks, the surface current distribution also confirms the proper functioning of the feeding network, verifying once again the frequency multiplexed structure achieved. Figure 11 starts by presenting the surface current distribution at the operating frequencies of the dual-band antenna, 28 GHz and 38 GHz.

It can be easily observed that, for both frequencies, only the respective resonant monopole is being fed. The opposite monopole has no current flowing at all.

**Figure 12:**
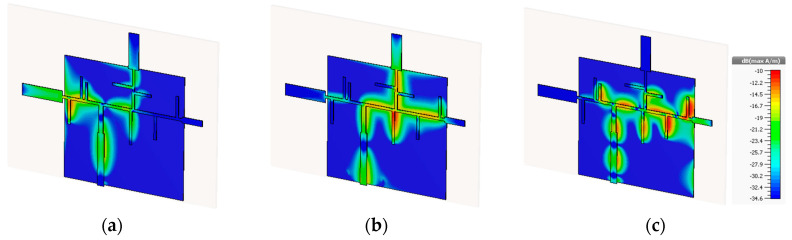
Surface Current Distribution at (**a**) 20 GHz, (**b**) 28 GHz and (**c**) 38 GHz.

Figure 12 exhibits the respective results for the tri-band structure, denoting the surface current distribution for the three operating frequencies.

Once again at each one of the resonant frequencies, only the respective monopole has any current through it, all the others remain not fed. With this, it is possible to state that the frequency multiplexing concept proposed allowed to isolate, in frequency, each radiating element, maintaining a single input feed and without requiring any additional filters.

**Figure 13:**
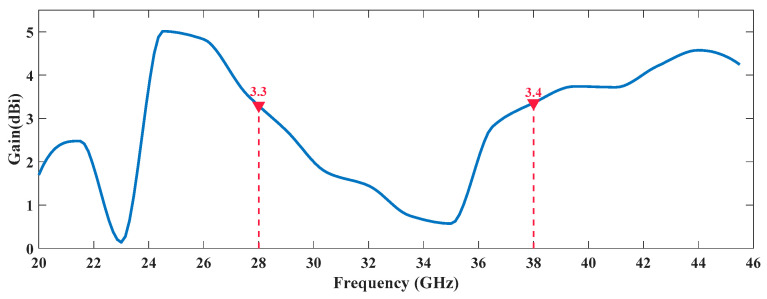
Gain Variation Over Frequency of the Dual-Band Antenna.

**Figure 14:**
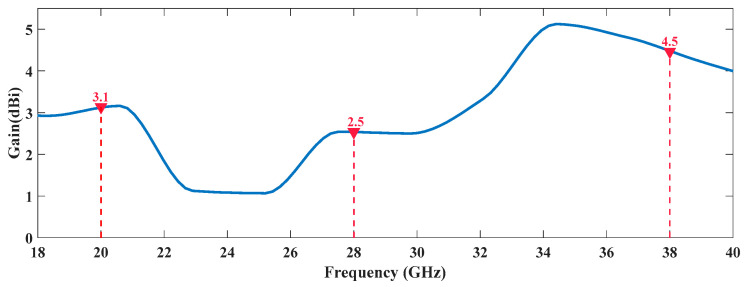
Gain Variation Over Frequency of the Tri-Band Antenna.

Figure 13 and Figure 14 show the gain variation of both prototypes over frequency, highlighting the respective values at the operating frequencies. It is possible to verify that, in the tri-band case, the gain values vary between 2.5 dBi and 4.5 dBi, in the frequencies of interest and they are mainly dependent on the antenna structure.

**Figure 15:**
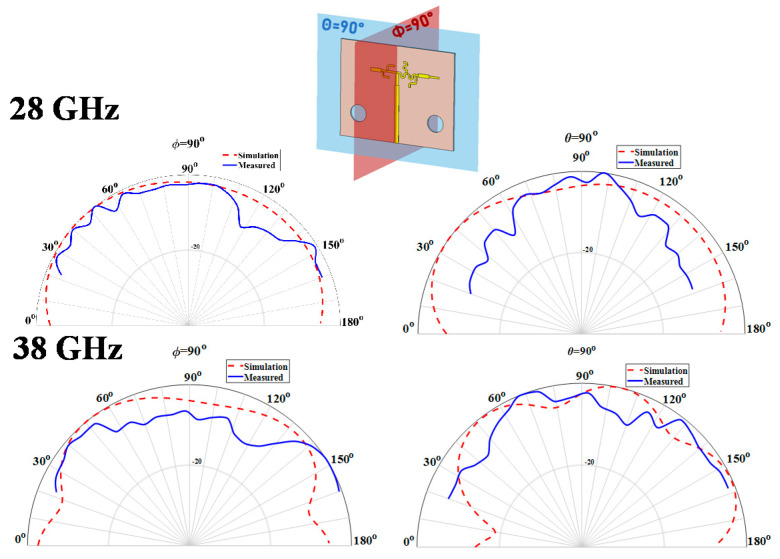
Simulated and Measured Normalized Radiation Pattern of the Dual-Band Antenna at 28 GHz and 38 GHz.

Regarding the gain of the antennas, in Figure 15 it is possible to observe the simulation and measured results of the two main radiation planes at both frequencies of interest, 28/38 GHz, for the dual band antenna.

As it is natural in very compact structures, particularly in multi-band antennas, the radiation patterns obtained seem a bit distorted from what was expected to be seen in monopoles. Apart from that, the dual-band antenna presents a maximum simulated gain of 3.3 dBi at 28 GHz, while at 38 GHz that value is 3.4 dBi.

**Figure 16:**
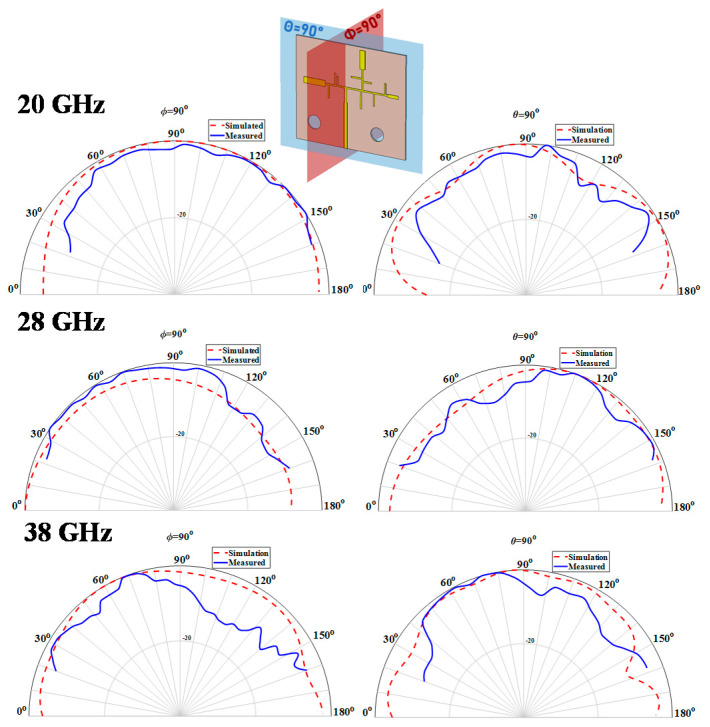
Simulated and Measured Normalized Radiation Pattern of the Tri-Band Antenna at 20/28/38 GHz.

When it comes to the tri-band antenna, the same two radiation planes are shown, but now for the three frequencies of interest. These results are presented in Figure 16 and a similar distortion in the radiation pattern shapes is observed. This radiating structure achieved a maximum simulated gain of 3.1, 2.5 and 4.5 dBi at 20, 28 and 38 GHz.

The main achievements of these structures are summarized and compared with the current state-of-the-art of dual and tri-band antennas in Table 5, analyzing the publication year (PY), the operating frequencies, bandwidth, size, scalability and the isolation in frequency factor.

Knowing that one of the main concerns of this letter is the microstrip frequency-selective feeding network able to maximize the efficiency as well as easing he interaction of a single antenna with multiple RF Frontends, Table 6 compiles some of the state-of-the-art works related to the frequency multiplexing argued, showing how it was obtained and the main characteristics of the antenna structure.

## 6. Conclusions

In this paper, a new multi-band antenna design concept is proposed, explained and proved through some practical prototypes. The main basis is the use of a feeding structure which ensure the impedance matching at a given operating frequency, isolating the structure at the remaining frequencies. When compared with an eventual alternative of a radiator with dual resonance, the option here presented, besides maximizing the efficiency, it avoids the usage of any bulky filters at the input.

This strategy was implemented in a dual-band and a tri-band prototypes, both suitable to integrate in IoT sensors. Individually these antennas present good impedance bandwidths for all frequencies of interest, since the smallest measured value is 400 MHz.

Regarding the antennas’ gain, and considering that these structures are based in monopoles, these values are also satisfactory, especially when allied to the efficiency values witnessed. More importantly, the proposed concept of producing a *n-band* antenna has proven not only to work, but it also assures higher efficiency values than what is commonly seen in the state-of-the-art for multi-band antennas.

## Figures and Tables

**Table 1:** Dual-Band Antenna’s Dimensions.

Parameter	Dimension
LMp28	1.30
WMp28	0.40
LMp38	1.24
WMp38	0.20
Lt	1.28
Wt	0.4115
Lti	1.60
Wti	0.40
Lin	1.30
Win	0.5228
x	10.50
y	6.00

**Table 2:** Tri-Band Antenna’s Dimensions.

Parameter	Dimension
LMp20	3.25
WMp20	0.86
LMp28	2.60
WMp28	0.80
LMp38	1.557
WMp38	0.43
Lti	2.00
Wti	0.33
Lin	3.90
Win	0.51
x	16.50
y	12.00

**Table 3:** Dual-Band S_11_ Parameter Characteristics.

	Measured Frequency Shift	Simulated Bandwidth	Measured Bandwidth
Around 28 GHz	680 MHz	1.48 GHz [27.22–28.7 GHz] 5.3%	2.55 GHz [27.54–30.09 GHz] 8.8%
Around 38 GHz	410 MHz	3.84 GHz [36.08–39.92 GHz] 10.1%	1.98 GHz [37.2–39.18 GHz] 5.2%

**Table 4:** Tri-Band S_11_ Parameter Characteristics.

	Measured Frequency Shift	Simulated Bandwidth	Measured Bandwidth
Around 20 GHz	330 MHz	0.88 GHz [19.56–20.44 GHz] 4.4%	0.62 GHz [20.05–20.67 GHz] 3%
Around 28 GHz	190 MHz	0.29 GHz [27.64–27.93 GHz] 1.1%	0.4 GHz [27.61–28.01 GHz] 1.4%
Around 38 GHz	1.5 GHz	4.03 GHz [34.60–38.62 GHz] 11%	2.16 GHz [35.45–37.61GHz] 5.9%

**Table 5:** State-of-the-Art of Multi-Band Antennas: Main Results.

[Ref.] PY	Antenna’s Description	Operating Frequencies	Bandwidth	Measurements	Scalability	Frequency Isolation
[2] 2015	Dual-Band Slot-Based	28/38 GHz	24–30 GHz 34–41 GHz	No	No	No
[4] 2016	Series-Fed Antenna Array	28/38 GHz	27.8–28.6 GHz 36–38.6 GHz	No	No	No
[5] 2015	SIW Antenna Array	28/38 GHz	0.32 GHz 1.9 GHz	No	No	No
[6] 2017	Annular Ring Slots	20/30 GHz	20.13–20.8 GHz 30–30.85 GHz	Yes	Yes	No
[7] 2018	Microstrip Patch	24.4/28/38 GHz	23.6–24.4 GHz 27.2–28.4 GHz 37.3–38.1 GHz	No	No	No
[8] 2019	Antenna Array with Defected Ground	29/37.4/41 GHz	28.5–29.7 GHz 36.7–38 GHz 40–42.1 GHz	No	No	No
[9] 2008	Printed Slot-Based Structures	2.4/5.2 GHz	2.48–3.02 GHz 3.81–5.65 GHz	Yes	Yes	No
[10] 2020	Aperture-Sharing Technique with a 4 Unit Linear Array and a Dipole	3.5/28 GHz	3.12–3.84 GHz 24.9–30.6 GHz	Yes	No	No
[11] 2019	Cavity-Backed Antennas using HMSIW Technique	4.6/5.48 GHz	91 MHz 20 MHz	Yes	Yes	No
		4.5/5/5.5 GHz	70 MHz 20 MHz 20 MHz	Yes	Yes	No
[13] 2004	Fork-Shaped Microstrip Monopole Antenna	860/2280 MHz	731–987 MHz 1498–3080 MHz	No	No	No
This Work	Monopole-Based with Frequency Selective Feeding Network	28/38 GHz	27.54–30.09 GHz 37.2–39.18 GHz	Yes	Yes	Yes
		20/28/38 GHz	20.05–20.67 GHz 27.61–28.01 GHz 35.45–37.61 GHz	Yes	Yes	Yes

**Table 6:** State-of-the-Art in Frequency Selective Antenna Structures: Main Results.

[Ref.] PY	Antenna’s Description	Central Frequencies	Frequency Isolation Technique	Feeding Network Complexity	Number of Ports/Element	Does it Require Additional Components?
[14] 2019	Ground Tapered Slot and Microstrip Feeders	2/32.5 GHz	Low-Pass Filter	High	2	Yes
[15] 2016	Antenna Array	3 GHz	3 dB directional couplers + Schiffman C sections + Filters and Power Dividers	Extreme	1	Yes
[16] 2017	Antenna Array	2.6 GHz	Directional Filter and Power Dividers	High	1	Yes
[17] 2020	Filtering Slot Antennas	3.5/5.2 GHz	*n*-mode resonators	Medium	2	No
		2.5/3.5/5.1 GHz				
This Work	Monopole-Based with Frequency Selective Feeding Network	28/38 GHz	Impedance Matching	Low	1	No
		20/28/38 GHz				
